# YOLOv11-LADC: A Lightweight Detection Framework for Micro–Nano Damage Precursors in Thermal Barrier Coatings

**DOI:** 10.3390/nano15241878

**Published:** 2025-12-14

**Authors:** Cong Huang, Xing Peng, Feng Shi, Ci Song, Hongbing Cao, Xinjie Zhao, Hengrui Xu

**Affiliations:** 1 College of Intelligence Science and Technology, National University of Defense Technology, Changsha 410073, China; huangc_2003@nudt.edu.cn (C.H.); shifeng@nudt.edu.cn (F.S.); songci@nudt.edu.cn (C.S.); hb_c@nudt.edu.cn (H.C.); zhaooxinjie@163.com (X.Z.); xuhengrui@nudt.edu.cn (H.X.); 2 National Key Laboratory of Equipment State Sensing and Smart Support, Changsha 410073, China; 3 Hunan Provincial Key Laboratory of Ultra-Precision Machining Technology, Changsha 410073, China

**Keywords:** deep learning, LSKA attention mechanism, deformable convolution, micro–nano damage precursors, data augmentation

## Abstract

Performance breakthroughs and safety assurance of aerospace equipment are critical to the advancement of modern aerospace technology. As a key protective system for the hot-end components of aeroengines, thermal barrier coatings (TBCs) play a vital role in ensuring the safe operation of aeroengines and overall flight safety. To address the core detection technology challenge for micro–nano damage precursors in aerospace TBCs, this study proposes an enhanced detection framework, namely YOLOv11-LADC. Specifically, the framework integrates the LSKA attention mechanism to construct the C2PSA-LA module, thereby enhancing the detection capability for micro–nano damage precursors and adaptability to complex small-sample datasets. Additionally, it introduces deformable convolutions (DeformConv) to build the C3k2-DeformCSP module, which dynamically adapts to the irregular deformations of micro–nano damage precursors while reducing computational complexity. A data augmentation strategy incorporating 19 transformations is employed to expand the dataset to 5140 images. A series of experimental results demonstrates that, compared with the YOLOv11 baseline model, the proposed model achieves a 1.6% improvement in precision (P) and a 2.0% increase in recall (R), while maintaining mAP50 and mAP50-95 at near-constant levels. Meanwhile, the computational complexity (GFLOPs) is reduced to 6.2, validating the superiority of the enhanced framework in terms of detection accuracy and training efficiency. This further confirms the feasibility and practicality of the YOLOv11-LADC algorithm for detecting multi-scale micro–nano damage precursors in aerospace TBCs. Overall, this study provides an effective solution for the intelligent, high-precision, and real-time detection of multi-scale micro–nano damage precursors in aerospace TBCs.

## 1. Introduction

Within the realm of modern aerospace technology, breakthroughs in flight equipment performance and safety assurance remain pivotal drivers of technological advancement. As the core component of aircraft, the safety and reliability of aeroengines underpin a range of flight characteristics, including stability, maneuverability, and payload capacity [1]. Thermal barrier coatings (TBCs) form a critical protective system sprayed onto the hot-end components of aircraft engines. Its typical structure comprises a ceramic layer and a metallic bonding layer sprayed onto the substrate. The ceramic layer employs partially Yttrium-stabilized Zirconia (ZrO_2_-7,8% Y_2_O_3_, 7–8% YSZ), effectively blocking inward heat transfer from high-temperature combustion gases. The metallic bonding layer, composed of MCrAlY (where M is Ni, Co, or Ni/Co), ensures robust adhesion between the substrate and ceramic layer [2,3,4,5]. Thermal barrier coatings exhibit high thermal resistance, low thermal conductivity, corrosion resistance, and phase stability, providing effective thermal insulation while significantly enhancing engine efficiency and reliability [6]. However, operating under extremely harsh thermomechanical–chemical coupled environments, these coatings are prone to micro- and nano-scale damage such as cracking, spalling, sintering, and corrosion. These micro–nano damage precursors serve as early warning signs of coating failure, severely compromising operational safety. Failure to detect and maintain them in a timely manner is likely to cause premature coating failure, ultimately resulting in catastrophic component damage or even full aircraft accidents [7,8,9]. Therefore, efficient and precise detection and identification of TBC micro–nano damage precursors are crucial for ensuring the operational safety and reliability of high-end equipment.

Traditionally, micro- and nano-damage detection in TBCs has primarily relied on visual inspection and other conventional methods. Human observation can identify severely damaged, unusable TBCs. However, these traditional approaches heavily depend on the inspector’s experience, suffering from limitations such as time-consuming procedures, high subjectivity, and susceptibility to missed defects. They fail to meet the stringent demands of modern industrial standards. Existing non-destructive testing (NDT) techniques also struggle to meet the stringent requirements for detecting and evaluating precursors of micro- and nano-damage in TBCs. For instance, ultrasonic testing employs transducers to emit ultrasonic waves and detect internal micro- and nano-damage in specimens [10]. However, it is insensitive to micro- and nano-damage forms like voids and microcracks. Furthermore, this method requires applying coupling agents to the specimen surface, which can contaminate the TBC and make it difficult to apply for in situ, in-service NDT of TBCs. Eddy current testing is constrained by the material, conductivity, shape, and dimensions of the inspected specimen, leading to certain errors in the results [11]. Acoustic emission testing is a dynamic non-destructive testing method [12] that detects micro- and nano-damage by applying a specific load to the specimen surface. However, due to the presence of influencing factors in the applied load and the inability to determine whether the detected damage is a precursor to micro- or nano-damage formed after load application on the TBC surface, this method cannot be used for non-destructive testing of thermal barrier coatings.

In recent years, scholars have extensively researched methods for detecting precursors of micro- and nano-damage in TBCs of aeroengines using machine vision, computers, and artificial intelligence technologies. Particularly, the revolutionary achievements of convolutional neural networks (CNNs) in computer vision have provided novel solutions for detecting such precursors on TBC surfaces. By intelligently analyzing and classifying images of micro- and nano-damage precursors collected from TBCs, these approaches enable the intelligent identification and categorization of these precursors. Two-stage detectors, exemplified by Faster R-CNN [13], offer high accuracy but suffer from slow processing speeds. In contrast, single-stage detectors like the YOLO (You Only Look Once) series [14,15,16] and SSD [17] achieve an excellent balance between speed and accuracy, rendering them more suitable for industrial online inspection. Cui et al. [18] proposed SDDNet, enhancing texture variation handling through feature retention modules. It achieved 88.8% mAP on standard datasets like NEU-DET with notable real-time performance, though detection of small-scale micro- and nano-damage precursors remains insufficient. Jain et al. [19] explored GAN-generated synthetic data augmentation, boosting classification sensitivity to 95.33% on the NEU-CLS dataset. This demonstrates that data augmentation can compensate for insufficient annotations, but it suffers from training complexity and poor scalability and fails to achieve accurate damage localization. Liu et al. [20] designed MVFNet, integrating a spatial feature pyramid network, achieving 73.5% AP on the self-built TBD blade dataset. It efficiently processes high-resolution images, but its generalization capability requires further validation.

The YOLO series algorithms are efficient deep learning-based object detection techniques that regard object detection as a regression problem, offering high real-time performance and accuracy [21,22]. Zhang, Daiwei et al. [23] trained a network model based on YOLOv3 to automatically detect faulty images of aeroengine blades, which exhibited excellent blade damage recognition performance yet lacked quantitative indicators. Li et al. [24] proposed DDSC-YOLOv5s, which optimizes feature extraction through a deformable convolutional network, resulting in an mAP@50 of 83.8%. Detection accuracy improved by 1.9%, but computational complexity increased by 7.9%. To address the difficulties in detecting aeroengine blades under complex conditions and associated safety hazards, Li et al. [25] proposed an improved YOLOv7 model by embedding a channel attention module and replacing CIoU with Alpha-GIoU. This achieved an mAP of 96.1% with strong real-time performance, though it exhibited high dependency on image quality.

To overcome the challenges of error-prone and time-consuming detection processes while enhancing accuracy and speed, researchers have explored various approaches. For instance, Liao et al. [26] developed an optimized YOLOv5 model with a Bi-FPN architecture to enhance detection precision and reduce processing time. Wang et al. [27] introduced DBFF-YOLOv4, employing dual-backbone feature fusion and data augmentation techniques like cropping and flipping to significantly boost recall, though a 7% false positive ratio remains a challenge. Chen et al. [28] introduced an attention-augmented path aggregation neck to enhance the ability to extract salient differential features, achieving a 4.1% improvement in mAP.

However, the direct application of the general-purpose YOLOv11 to the detection of micro–nano damage precursors in TBCs remains confronted with several key challenges: First, critical micro–nano damage precursors (e.g., microcracks) typically occupy only a minute fraction of image pixels, rendering them recalcitrant to detection by YOLOv11. Second, the ultra-small scale of micro–nano damage precursors necessitates models to possess specialized micro–nano-scale feature capture capabilities. Third, the low contrast between micro–nano damage precursors and the coating background frequently results in elevated false-negative rates.

To address the high false-negative rates, excessive model complexity, and inadequate adaptability to micro–nano scales encountered in TBC micro–nano damage precursor detection, this study proposes YOLOv11-LADC—an improved detection model tailored for TBC micro–nano damage precursors, leveraging an adaptive sampling mechanism and attention enhancement. The core objective is to construct a specialized network architecture suited for the detection of small-sample, micro–nano-scale, and low-contrast defects, thereby achieving high-precision detection and effective feature representation of micro–nano damage precursors. Additionally, the model adopts a lightweight design to ensure compatibility with resource-constrained complex industrial environments. Specifically, the YOLOv11 architecture is enhanced through the following modifications: (1) Integration of the Large Separable Kernel Attention (LSKA) mechanism into the backbone network to strengthen the model’s representation of micro–nano-scale features in damage precursors, enhance its focus on target regions, and suppress interference from complex device surface backgrounds; (2) Incorporation of the C3k2-DeformCSP module for the construction of a Deform-Neck. This module adapts to the irregular deformations of multi-scale micro–nano damage precursors via a dynamic sampling mechanism, improving the robustness of feature extraction and expanding the receptive field. Concurrently, it reduces computational complexity in line with lightweight design principles, enabling the model to be applied in resource-constrained complex industrial environments; (3) Employment of data augmentation techniques on the training dataset to expand the multi-scale micro–nano damage precursor image dataset of TBCs. This mitigates errors induced by data category imbalance and addresses the bottleneck of insufficient sample data acquisition during model training.

The structure of this paper is as follows: Section 2 introduces the original YOLOv11 model architecture, the improved YOLOv11-LADC network architecture, and the network structures and underlying principles of each enhanced module; Section 3 details the experimental setup, data augmentation processing, and selection of evaluation metrics; Section 4 presents a series of training experiments based on the improved YOLOv11-LADC model, including model comparisons, ablation studies, and loss comparisons during training and validation, along with analysis of detection results; Section 5 concludes with a summary of this research.

## 2. Proposed Method

### 2.1. Model Network Architecture

**Figure 1:**
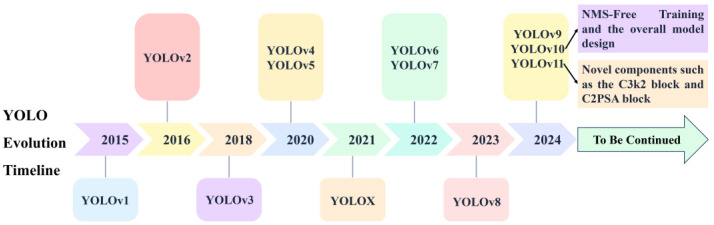
YOLO evolution timeline.

The YOLO series models constitute a family of efficient and real-time object detection algorithms [7]. First proposed in 2015, they have undergone multiple rounds of iterative optimization. The evolution timeline of the YOLO series models is illustrated in Figure 1.

**Figure 2:**
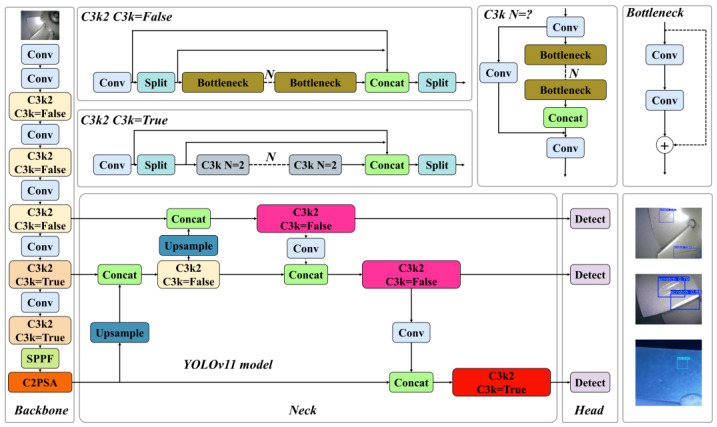
YOLOv11 model network architecture.

As a typical single-stage detection framework, the YOLO series occupies a pivotal position in computer vision due to its outstanding real-time inference performance and continuously improved detection accuracy. This algorithm system performs excellently in object detection tasks and has also achieved notable research results in multiple visual subfields such as image classification, semantic segmentation, moving object tracking, and human pose estimation. Currently, it has been widely applied in both industry and academia. Released at the YOLO Vision 2024 (YV24) conference, YOLOv11 represents a major leap forward in object detection technology [29]. This paper adopts the YOLOv11n model as the basic model, and the overall structure of the original model is shown in Figure 2.

The YOLOv11 model comprises Backbone, Neck, and Head components.

The backbone network is mainly composed of Convolution (Conv) modules, C3k2 modules, SPPF modules, and C2PSA modules. The Conv module includes 2D convolution layers, Batch Normalization (BN) layers, and activation functions, undertaking basic feature extraction. YOLOv11 introduces a novel C3k2 mechanism, which takes a parameter c3k to realize dynamic optimization of shallow and deep networks. The SPPF module performs multi-scale pooling operations, minimizing computational complexity while maximizing the retention of key information during downsampling. The C2PSA module embeds a multi-head attention mechanism into the basic C2 module architecture. In the Head network, the DWConv module only processes spatial-dimension convolutions without inter-channel convolutions. It supplements channel information via 1 × 1 pointwise convolution, efficiently reducing parameter quantity and computational complexity while compensating for the lack of cross-channel information integration capability.

**Figure 3:**
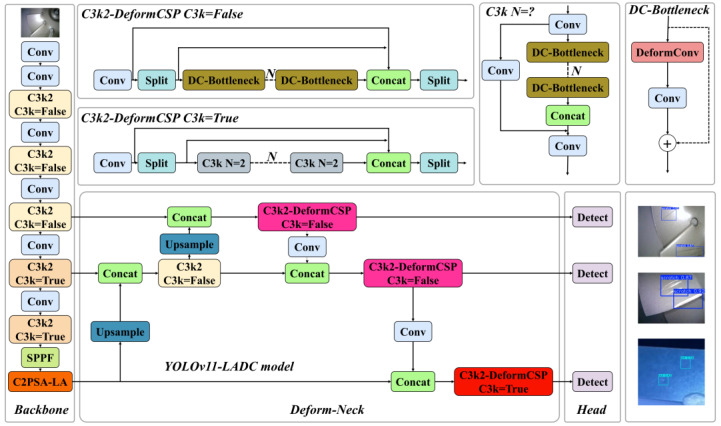
Improving the network architecture of the YOLOv11-LADC model.

The YOLOv11 model shows significant performance improvements among the YOLO series, mainly reflected in stronger adaptability and better scalability. However, it exhibits certain limitations when tackling practical, task-specific object detection scenarios [30]. Specifically, in the application of detecting micro–nano damage precursors in aerospace TBCs, prevalent challenges include small target scales, low contrast between targets and backgrounds, high false positive/negative rates, and model overfitting. To address these issues, this study proposes an improved detection model—YOLOv11-LADC—tailored for micro–nano damage precursors in TBCs, integrating an adaptive sampling mechanism and attention enhancement. The model achieves enhanced detection performance and task adaptability through three key optimizations: Introducing the LSKA attention mechanism into the backbone network to strengthen micro–nano-scale feature extraction and suppress interference from complex backgrounds; Incorporating deformable convolutions into the neck network to dynamically adapt to the irregular morphologies of damage precursors and improve the robustness of feature extraction; Employing nineteen image transformation techniques for dataset augmentation, which mitigates sample scarcity and class imbalance while enhancing the model’s generalization capability. This research establishes an efficient and accurate detection system for micro–nano damage precursors in aerospace TBCs, with the optimized network architecture illustrated in Figure 3.

To address the aforementioned issues, enhance the detection accuracy of the original model, and reduce computational complexity, this study implements two improvements to the YOLOv11 network architecture: First, the LSKA attention mechanism is integrated with the Backbone, and the C2PSA-LA module is proposed to replace the original C2PSA module. This modification boosts the model’s ability to detect micro–nano damage precursors and improves its overall detection and classification performance. Second, considering that introducing attention mechanisms may increase model complexity, and given the small sample size of the aeroengine thermal barrier coating micro–nano damage precursor image dataset—where complex models show poor generalization, risk overfitting to training data, and face challenges in detecting irregularly deformed micro–nano damage precursors—this study specifically introduces DeformConv (deformable convolution with adaptive spatial sampling and reduced computational complexity). The C3k2-DeformCSP module is constructed, which uses a dynamic sampling mechanism to adapt to the irregular deformations of micro–nano damage precursors. It enhances the robustness of feature extraction, expands the receptive field, and reduces model complexity without sacrificing network accuracy.

### 2.2. C2PSA-LA Module Structure

In optimizing detection models for micro–nano damage precursors in aeroengine thermal barrier coatings, enabling the model to adapt to micro–nano damage precursors of different scales is a key objective—especially for identifying damage types with vastly different scales, such as scratches and micro-pores. This module integrates large-kernel convolutions with the LSKA attention mechanism, which expands the receptive field and enhances global modeling capability. It thereby suppresses complex background interference and achieves multi-scale adaptation for micro–nano damage precursors (e.g., microcracks and pores) of varying sizes [31]. The core calculation formulas for the LSKA attention mechanism are as follows:

Given an input feature map $F\in R^{C\times H\times W}$ (where *C* denotes the number of input channels, and *H* and *W* represent the height and width of the feature map, respectively), depthwise convolution is computed as:

(1) $${\bar{Z}}^{C}=\sum_{H,W}\;W_{(2d-1)\times 1}^{C}*\left(\sum_{H,W}\;W_{1\times (2d-1)}^{C}*F^{C}\right)$$

In the equation, $W^{C}$ denotes the weights of the deep convolutional kernel, where the superscript *C* indicates independent computation per channel. *d* represents the stride rate, and ∗ denotes the convolution operation. This deep convolution computation is employed to capture local spatial information.

Perform deep dilated convolution computation:

(2) $$Z^{C}=\sum_{H,W}\;W_{\left\lfloor \frac{k}{d}\right\rfloor \times 1}^{C}*\left(\sum_{H,W}\;W_{1\times \left\lfloor \frac{k}{d}\right\rfloor }^{C}*{\bar{Z}}^{C}\right)$$

In the equation, *k* denotes the size of the original large convolution kernel, and $\left\lfloor \frac{k}{d}\right\rfloor$ represents the floor operation. The dilated deep convolution is responsible for capturing the global spatial information of the deep convolution output ${\bar{Z}}^{C}$.

Channel dimension compression via 1×1 convolution generates attention weights:

(3) $$A^{C}=W_{1\times 1}*Z^{C}$$

In the equation, $W_{1\times 1}$ denotes a 1×1 convolution kernel, which computes the attention weight $A^{C}$ through convolution with $Z^{C}$.

Finally, feature enhancement is achieved via the Hadamard product:

(4) $${\bar{F}}^{C}=A^{C}⊗F^{C}$$

In the equation, ⊗ denotes the Hadamard product operation, which is used to emphasize or suppress different parts of the feature map, thereby focusing on important information.

**Figure 4:**
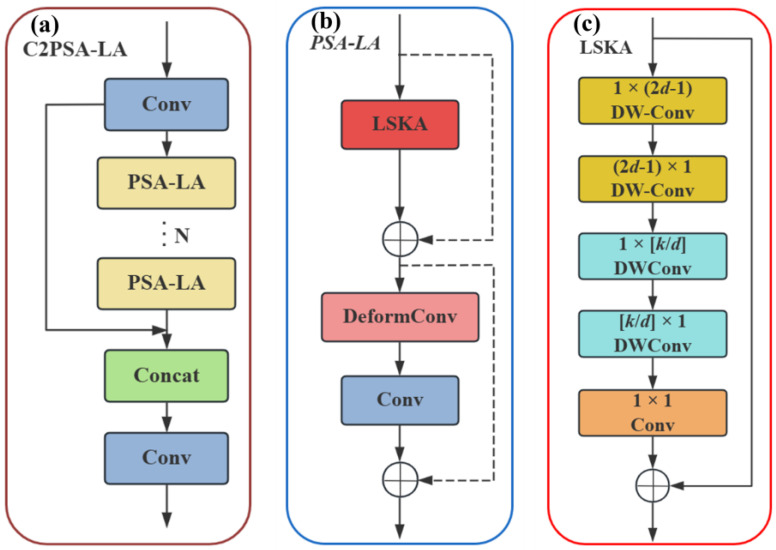
Schematic diagram of the C2PSA-LA module structure. (**a**) C2PSA-LA; (**b**) PSA-LA; (**c**) LSKA.

The structural diagram of the C2PSA-LA module is shown in Figure 4.

For the detection scenario of micro–nano damage precursors with complex multi-scale and small-sample characteristics in this study, we innovatively integrate the LSKA attention mechanism with the Backbone, proposing the C2PSA-LA module to replace the original C2PSA module. Large-kernel depthwise separable convolutions are introduced to expand the receptive field, achieving modeling capabilities close to global attention and enabling more effective capture of global contextual information. This enhances the model’s ability to detect multi-scale micro–nano damage precursors and handle complex small-sample datasets, while strengthening its focus on micro–nano damage precursor regions [32].

### 2.3. C3k2-DeformCSP Module Structure

This module introduces the DeformConv deformable convolution to enhance the model’s detection capability for multi-scale complex micro–nano damage precursors while implementing a lightweight design to address the issue of small- sample dataset capacity. The schematic diagram of the C3k2-DeformCSP module structure is shown in Figure 2. The calculation formula for deformable convolution is as follows:

(5) $$y\left(p_{0}\right)=\sum_{p_{n}\in R}\;w\left(p_{n}\right)*x\left(p_{0}+p_{n}+\Delta p_{n}\right)$$

In the formula, $w\left(p_{n}\right)$ denotes the weight at the corresponding position on the convolution kernel, and $\Delta p_{n}$ represents the offset relative to *x*. This allows deformable convolution to modify the sampling region of the input feature map.

Here, $p_{n}$ denotes the offset of each coordinate point relative to the center point, which can be expressed as Formula (6):

(6) R={(−1,−1),(−1,0),…,(0,1),(1,1)}

**Figure 5:**
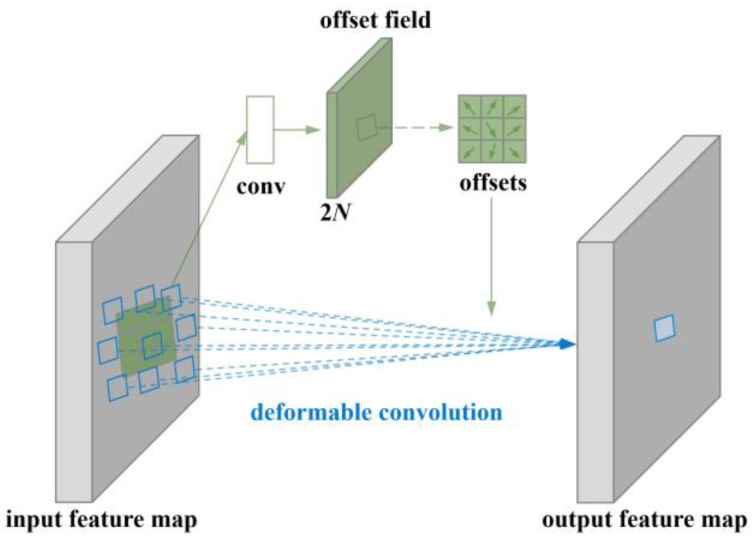
Schematic diagram of deformable convolution.

The schematic diagram of deformable convolution is presented in Figure 5.

To ensure the model possesses robust detection capabilities for multi-scale complex micro–nano damage precursors while maintaining generalization performance trained on small datasets based on the principle of reducing computational complexity. This module introduces DeformConv, a deformable convolution technique. Its adaptive sampling mechanism dynamically adjusts the shape and size of the receptive field to match variations in the shape and position of multi-scale micro–nano damage precursors, achieving significant detection performance for small and irregular micro–nano damage precursor targets [33]. Additionally, based on lightweight design principles, it reduces computational complexity and inference time, preserves generalization to prevent overfitting, and accurately captures key feature information in micro–nano damage precursor textures.

Through such targeted modular design, a network architecture suitable for detecting micro–nano damage precursors in aeroengine thermal barrier coatings is constructed.

## 3. Experimental Configuration

### 3.1. Experimental Environment Setup

This experiment uses the Windows 11 operating system as the experimental platform, adopts the PyTorch(version 2.3.1) deep learning framework for algorithm model construction, and utilizes the Anaconda tool to create an independent virtual environment to ensure the isolation of the experimental environment. Experimental parameter settings are shown in Table 1.

### 3.2. Dataset Selection

**Figure 6:**
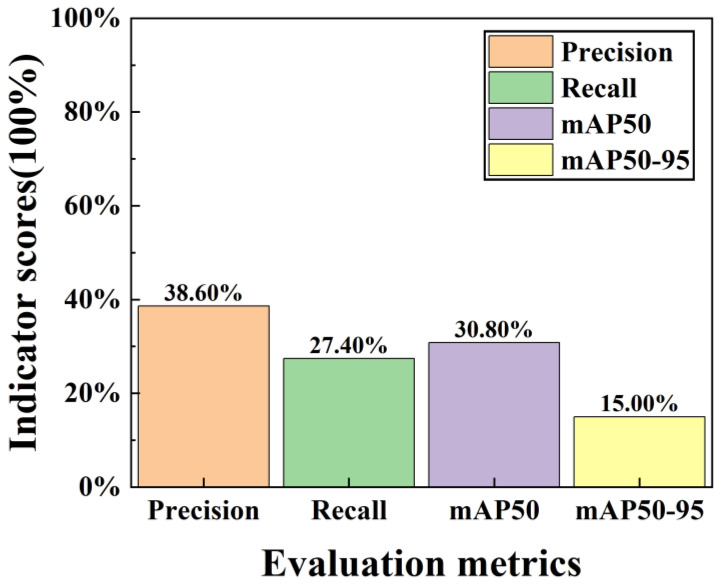
Training Metric Score Plot for Datasets Without Data Augmentation.

This study utilizes the Aeroengine-defect-detect micro–nano defect precursor dataset, which contains only 257 training images. This limited sample size may result in insufficient feature extraction during model training. By training the YOLOv11 baseline model on this dataset, the evaluation metrics are shown in Figure 6.

Analyzing Figure 6, the dataset before data augmentation achieved Precision, Recall, mAP50, and mAP50-95 of 38.60%, 27.40%, 30.80%, and 15.50%, respectively. Due to the insufficient sample size of the original dataset, the features extracted during training were inadequate, resulting in limited detection performance that rendered the dataset unusable for subsequent training. To address this issue, data augmentation was employed to expand the dataset from a limited number of samples. This approach enhances the model’s generalization capability and reduces overfitting [34]. By controlling the types of data augmentation, sample balance was achieved, minimizing errors caused by data imbalance and improving the equilibrium of category distribution.

**Figure 7:**
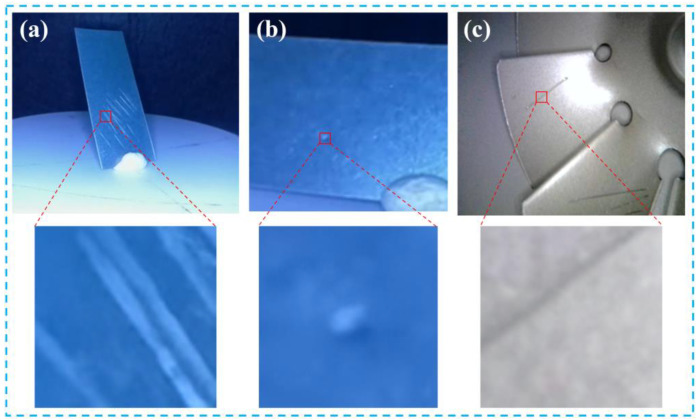
Enlarged Annotated Diagram of Micro–Nano Damage Precursors in Thermal Barrier Coatings; (**a**) Scratches 1; (**b**) Stains; (**c**) Scratches 2.

The original micro–nano damage precursors are classified into four types: surface scratches, stains, creases, and damage. Figure 7 displays enlarged and annotated images of micro–nano damage in selected TBCs.

**Figure 8:**
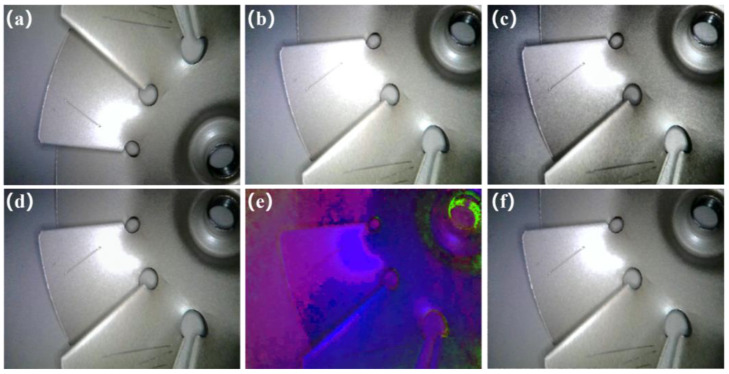
Partially data-enhanced images: (**a**) Vertical flipping; (**b**) Sharpening; (**c**) Adaptive histogram equalization; (**d**) Gaussian noise; (**e**) Color space transformation; (**f**) Elastic transformation.

LabelImg software(version 1.8.6) was used to annotate the original images with micro–nano damage precursor labels, and the annotation files provide detailed information on the location, size, and category of each precursor. Code was developed to perform batch data augmentation on the original images, with simultaneous expansion of annotation files. The augmentation methods include three categories: geometric transformations, color transformations, and special transformations. A total of 19 augmentation types were employed in this study, namely Gaussian noise transformation, translation along the positive x-axis, translation along the positive y-axis, color space transformation, adaptive histogram equalization (AHE) transformation, elastic deformation, horizontal flipping, gamma correction, Gaussian blur, contrast adjustment, median filtering, brightness modification, hue adjustment, saturation adjustment, sharpening, cropping, vertical flipping, histogram equalization, and perspective transformation. These aim to simulate variations encountered in real-world detection scenarios (such as uneven lighting, viewing angle shifts, and surface reflections). rather than introducing unrealistic structural artifacts. Partial effect diagrams are shown in Figure 8. To enhance the representativeness of the dataset, this study utilized the aforementioned data augmentation techniques, applying all specified transformations to 257 precisely annotated original images. Each transformation generated a new image distinct from the original while preserving micro–nano damage precursor features, and the corresponding label files were updated synchronously. Ultimately, this approach expanded the total dataset from 257 original images to 5140 augmented images, which broadened the distribution of training data and mitigated the risk of overfitting.

The dataset of thermal barrier coating damage precursor samples is inherently limited in volume. To address this limitation—and to complement the improved YOLOv11 model (which incorporates attention mechanisms and deformable convolutions)—additional training data is required to prevent underfitting and ensure the model fully learns the target features. Consistent with literature on damage detection in complex industrial settings, the dataset was partitioned into training, validation, and test subsets using an 8:1:1 ratio [24]. Specifically, both the validation and test subsets (each accounting for 10% of the total dataset) contained 514 damage precursor images. This partitioning strategy ensures reliable evaluation of model performance and statistical metrics while optimizing training efficiency. To maintain category balance and annotation quality, comparative analyses were conducted on the sample category distribution and label consistency before and after data augmentation. The augmentation techniques effectively increased the number of samples for less frequent micro–nano damage precursor categories, achieving near-perfect category balance. Additionally, randomly selected images and their corresponding labels underwent manual review to confirm 100% label consistency.

### 3.3. Evaluation Indicators Selection

This paper evaluates the model using five metrics: GFLOPs, Precision, Recall, mean Average Precision at 50% (mAP50), and mAP50-95 [35]. The formulas for each metric are as follows.

(1) Precision

Precision denotes the proportion of correctly classified samples out of the total number of samples, as shown in Formula (7).

(7) $$Precision=\frac{TP}{TP+FP}$$

(2) Recall

Recall denotes the proportion of correctly predicted positive samples among all actual positive samples, as shown in Formula (8).

(8) $$Recall=\frac{TP}{TP+FN}$$

In the formula, *TP* denotes the number of positive samples predicted as positive, *TN* denotes the number of negative samples predicted as negative, *FP* denotes the number of negative samples predicted as positive, and *FN* denotes the number of positive samples predicted as negative.

(3) Mean Average Precision (mAP)

Average Precision (AP) comprehensively evaluates the trade-off between Precision and Recall by using the area under the Precision-Recall curve as the metric, as shown in Formula (9).

(9) $$\mathrm{AP}=\int_{0}^{1}p\left(r\right)dr$$

In the formula, p(r) denotes the precision as a function of recall r.

The mean average precision (mAP) is the average precision across multiple categories, comprehensively reflecting precision, recall, and the average precision value. mAP50 denotes the mAP value at an IOU threshold of 50%. mAP50-95 represents the average of mAP values across the IOU threshold range from 50% to 95%. This metric provides a more accurate assessment of performance across different IOU thresholds, as shown in Formula 10.

(10) $$mAP=\frac{AP}{num_{classes}}$$

Here, $num_{classes}$ denotes the number of categories in the target task. The mAP value ranges from [0, 1], with values closer to 1 indicating better detection performance of the model.

(4) Computational Power (GFLOPs)

GFLOPs represents the number of billion floating-point operations executed per second. It serves as a key metric for measuring the complexity of deep learning models. Higher GFLOPs indicate a greater computational workload, signifying increased model complexity.

(11) $$O\left(\sum_{i=1}^{n}\;\;K_{i}^{2}*C_{i-1}^{2}+\sum_{i=1}^{n}\;\;M^{2}*C_{i}\right)$$

In the equation, *K_i_* denotes the size of the convolutional kernel for layer *i*, *C_i_* represents the number of output channels for layer *i*, *C_i_*_−1_ indicates the number of input channels for layer *i*, *M* is the spatial size of the feature map, and *n* is the number of layers in the network.

## 4. Result and Analysis

### 4.1. Series of Quantitative Network Analyses

**Figure 9:**
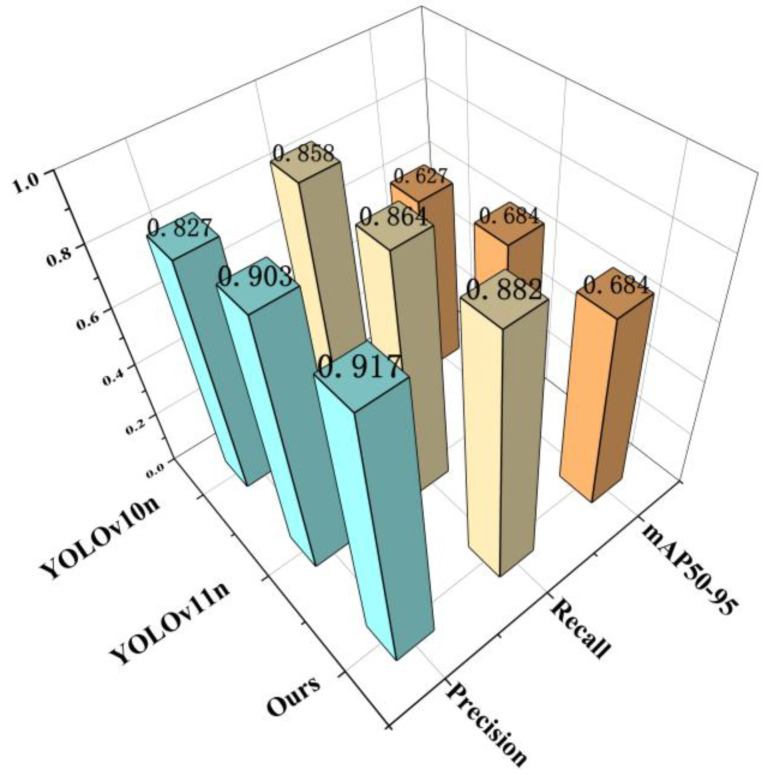
Comparison of metric scores for YOLO series models.

To evaluate the effectiveness of the improved algorithm proposed in this paper, we selected widely used object detection algorithms from the YOLO series for quantitative comparative experiments, including YOLOv10 [36], YOLOv11, and the YOLOv11-LADC designed herein. Evaluation metrics include GFLOPs, Precision, Recall, mAP50, and mAP50-95. Quantitative analysis results of the networks are shown in Table 2 and Figure 9.

Analysis of Table 3 and Figure 9 shows that lower GFLOPs indicate lower computational complexity. The computational complexity ranking of the models is YOLOv10n < YOLOv11n < YOLOv11-LADC. Thus, the improved YOLOv11-LADC model designed in this study has the lowest computational complexity, making it suitable for inference on small-sample datasets and low-power devices.

Bolded content indicates the optimal metric value under the same indicator. Moreover, the improved YOLOv11-LADC model outperforms YOLOv10n and YOLOv11n in precision by 9.8% and 1.6%, respectively, and in recall by 2.4% and 2.0%, respectively. It exceeds YOLOv10n in mAP50 and mAP50-95 by 3.1% and 8.3%, respectively, while being nearly identical to YOLOv11n. Considering both detection accuracy and computational complexity, YOLOv11 is selected as the baseline model for optimization in this study.

The proposed YOLOv11-LADC model maintains high precision and recall while achieving lightweight complexity. It has lower overall computational load and fewer parameters than the original baseline model, making it suitable for intelligent identification of micro/nano-damage precursors in thermal barrier coatings.

### 4.2. Melting Experiment

To intuitively analyze the impact of different improved modules on model evaluation metrics, we conducted a series of ablation experiments based on the YOLOv11n model to verify the effectiveness of the optimized network architecture proposed in this paper [37,38]. YOLOv11n was used as the baseline model to train the Aeroengine micro–nano damage precursor dataset, yielding detection evaluation metrics of the baseline model as the experimental benchmark. First, we improved the C2PSA module in the backbone network by introducing the LSKA attention mechanism and proposing the C2PSA-LA module to replace the original C2PSA module. The model with this improvement was trained on the micro–nano damage precursor dataset to obtain detection metrics of the preliminarily optimized model. Similarly, further improvements were made by introducing DeformConv deformable convolutions into the Neck network, and the improvement effect was verified, ultimately resulting in the YOLOv11-LADC network model designed in this paper.

To validate the effectiveness of DeformConv deformable convolutions selected in this paper, other convolutional modules were introduced into the Neck network for model training, and the resulting detection results of micro–nano damage precursors are shown in Table 3.

As shown in Table 4 are the detection results of introducing DSConv [39], AKConv [40], DWConv [41], and DeformConv [33] modules into the Neck network, respectively. DS-Neck and DW-Neck have a GFLOPs of 5.9, indicating the lowest computational load among these modules, but their detection performance after training is worse than the original model. In contrast, the Deform-Neck module designed in this paper is the most effective: its Precision is 1.3% higher than the original model and 3.9% and 3.3% higher than DS-Neck and DW-Neck, respectively. Although the improved Deform-Neck module has the same Precision as AK-Neck, its Recall is increased by 3.0%, and mAP50 and mAP50-95 are improved by 1.5% and 4.7%, respectively. The improved Deform-Neck has a Recall 0.8% higher than the original model and 4.4% higher than DS-Neck; although its Recall is slightly lower than DW-Neck, it significantly outperforms DW-Neck in the other three metrics. Compared with the DeformConv module, other modules show insignificant improvement or even decline in Precision, Recall, mAP50, and mAP50-95; while after introducing DeformConv, YOLOv11′s Precision and Recall are increased by 1.3% and 0.8%, respectively, mAP50 slightly decreases but remains basically stable, and mAP50-95 remains unchanged. Simultaneously, the integration of DeformConv yielded significant improvements in both precision and recall. Notably, mAP50 and mAP50-95 remained largely consistent with the baseline levels, with no appreciable increase in the false positive rate. This finding indicates that while DeformConv enhances the model’s capability to capture irregular micro–nano damage features, it does not substantially elevate the risk of misclassifications. Additionally, the GFLOPs metric decreased by 0.1, which reduces computational complexity and shortens inference time. This optimization achieves a lightweight design that addresses the limitation of small-sample datasets while preserving the model’s generalization ability to mitigate the risk of overfitting.

**Figure 10:**
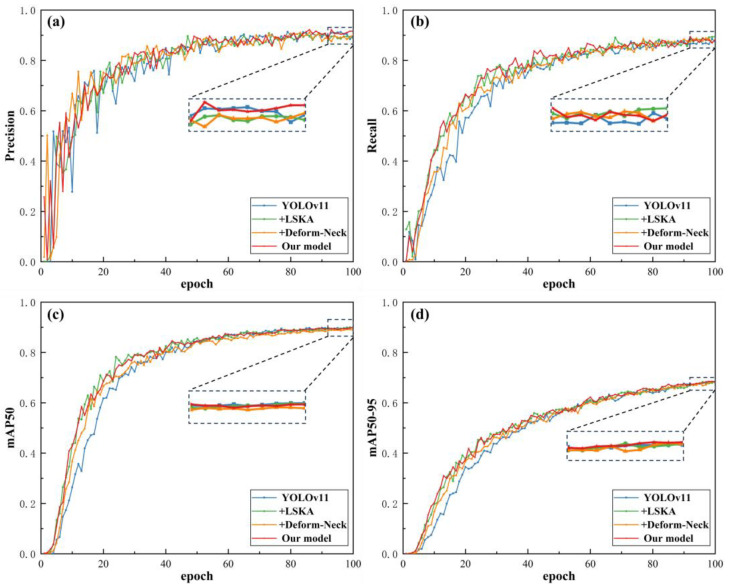
Module combination effectiveness ablation experiment results. (**a**) Precision; (**b**) Recall; (**c**) mAP50; (**d**) mAP50-95.

Based on the above comparative validation experiments on different convolutional modules, this paper further conducts ablation experiments to verify the effectiveness of combining the improved C2PSA-LA module and C3k2-DeformCSP module, with the experimental results shown in Table 4 and Figure 10.

Analysis of the experimental results in Table 4 shows that compared with the YOLOv11 base network, introducing the improved C2PSA-LA module decreases Precision by 1.7% but increases Recall by 3.6%. Moreover, the model’s GFLOPs are significantly lower than the original model (a decrease of 0.4), reducing model complexity and enabling more accurate capture of key feature information in micro–nano damage precursor textures. After introducing the improved C3k2-DeformCSP module, Precision increases by 1.3% and Recall by 0.8%. Combining these two improved modules into the model forms the YOLOv11-LADC model designed in this paper. After training on the Aeroengine micro–nano damage precursor dataset, this model achieves a 1.4% increase in Precision and a 2.1% increase in Recall compared with the original baseline YOLOv11 model. The 0.1 decrease in GFLOPs indicates the final model has lower structural complexity than the original. For detecting two types of micro–nano damage precursors (surface scratches and damage), the model improves AP by 0.8% for scratches and 3.8% for damage, showing significant effectiveness. Based on the above ablation experiment results, the structural improvement scheme in this paper greatly enhances detection performance while achieving model lightweighting to address small-sample dataset capacity issues, ensuring model generalization to prevent overfitting and making the YOLOv11 network more suitable for target detection tasks on the aeroengine thermal barrier coating micro–nano damage precursor dataset.

### 4.3. Model Loss Comparison

**Figure 11:**
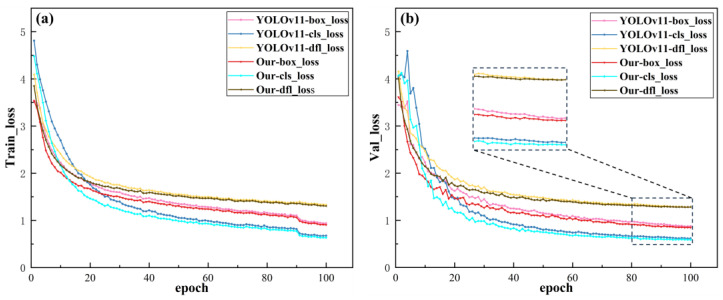
Comparison chart of model loss. (**a**) Train_loss; (**b**) Val_loss.

To validate the effectiveness of the proposed YOLOv11-LADC network architecture for model convergence and confirm that the model does not overfit to the augmented data, Figure 11 presents loss diagrams for both the training and validation processes.

Analysis of the model loss comparisons presented in Figure 11 indicates that the proposed YOLOv11-LADC model demonstrates highly synchronized and stable decreasing trends in both training and validation losses throughout the entire training process. It outperforms the original baseline model across three key loss metrics: bounding box regression loss (box_loss), class classification loss (cls_loss), and distribution feature loss (dfl_loss). The loss curves start with relatively high initial values, gradually decrease as training iterations progress, and converge to a stable state in the later training stages. After convergence, the numerical difference between the training and validation losses is minimal. This observation implies that the model’s performance on unseen validation data closely aligns with its performance on the training dataset, thereby further validating its robust generalization capability. Furthermore, the training and validation loss curves for each category of the YOLOv11-LADC model exhibit a steeper descending trend compared to those of the original baseline model. This suggests that the proposed model holds significant advantages over the original baseline in terms of training efficiency and convergence rate.

### 4.4. Comparison of Model Detection Results

**Figure 12:**
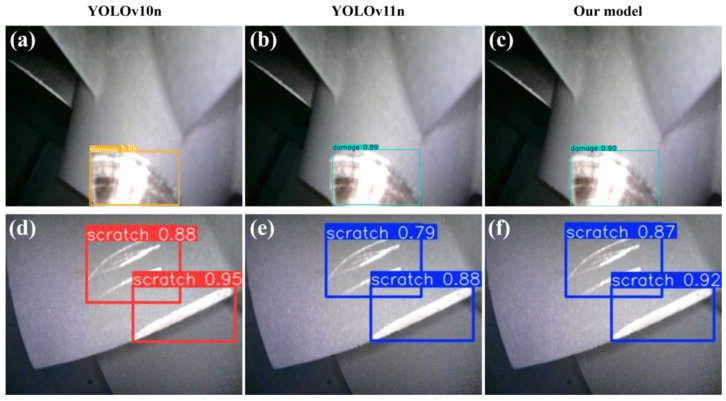
Comparison chart of model detection results. (**a**) YOLOv10n damage detection; (**b**) YOLOv11n damage detection; (**c**) Our model damage detection; (**d**) YOLOv10n scratch detection; (**e**) YOLOv11n scratch detection; (**f**) Our model scratch detection.

To systematically and intuitively validate the effectiveness of the proposed improved model, this study conducts qualitative analysis: two sample images that typically reflect the characteristics of thermal barrier coating micro–nano damage precursors are selected from the result dataset. These images display multiple key morphological and textural features present in practical scenarios, and Figure 12 shows the actual detection results of each model for these two images.

As shown in the detection result comparison of each model in Figure 12, although the YOLOv10n model achieves higher detection metrics for surface scratches (scratch) in Figure 12d than YOLOv11n and the model designed in this study, it incorrectly detects one damage as two in Figure 12a. Thus, the YOLOv10n model has significant errors in detecting damage-type micro–nano damage precursors. In contrast, the improved model designed in this study accurately identifies the correct forms of micro–nano damage precursors without over-detection or false detection. The detection confidence of the proposed model in Figure 12c,f is higher than that of YOLOv11n, and its bounding boxes are more precise.

In summary, the improved model designed in this study exhibits good detection accuracy for targets of different scales. This is because the LSKA attention mechanism and DeformConv convolution module introduced in the improved model can adapt to the shape and position variations in multi-scale micro–nano damage precursors, achieving excellent detection performance for small and irregular micro–nano damage precursor targets. Furthermore, this study adopts a lightweight design with a GFLOPs value of merely 6.2, enabling the model to operate in resource-constrained environments for the detection of micro–nano-scale damage precursors in complex thermal barrier coatings. Notably, the model exhibits robust performance when confronting complex backgrounds, micro–nano-scale targets, and irregularly shaped defects.

## 5. Conclusions

This study addresses the key challenges in detecting micro–nano damage precursors in TBCs, including high false-negative rates, excessive model complexity, and inadequate adaptability to micro–nano scales. To tackle these issues, an intelligent recognition network architecture tailored for micro–nano damage precursors in aerospace TBCs is designed, and a YOLOv11-LADC model is proposed based on adaptive sampling mechanisms and attention enhancement. Experimental validation confirms the effectiveness of the proposed model.

The YOLOv11-LADC model integrates the LSKA attention mechanism into its backbone network and adopts the C3k2-DeformCSP module to construct the Deform-Neck, with detailed descriptions of the overall architecture and optimization modules provided herein. This design enhances the model’s detection capability for micro–nano damage precursors while reducing computational complexity in line with lightweight design principles. During the experimental phase, relevant parameter settings are first outlined. Subsequently, 19 data augmentation techniques are employed to expand the dataset, thereby enhancing the model’s generalization capability and improving the balance of category distribution. Comparative experiments with other YOLO series models, ablation studies involving alternative convolutional modules, and efficacy tests for module integration are conducted. Compared to the YOLOv11 baseline model, the YOLOv11-LADC achieves a 1.6% increase in precision (P) and a 2.0% improvement in recall (R), while mAP50 and mAP50-95 remain largely unchanged. Specifically, the model attains precision, recall, mAP50, and mAP50-95 values of 91.7%, 88.2%, 89.7%, and 68.4%, respectively, demonstrating excellent detection performance for small targets and irregular micro–nano damage precursors. Finally, this study integrates training and validation loss curves with model detection results to comprehensively demonstrate the superiority of the proposed improved model in terms of detection accuracy and training efficiency. This further validates the feasibility and practicality of the YOLOv11-LADC algorithm for detecting multi-scale micro–nano damage precursors in aerospace TBCs.

## Figures and Tables

**Table 1:** Experimental Parameter Settings.

Hyperparameter	Parameter
Epochs	100
Batch size	16
Learning rate	0.01
Momentum	0.937
IOU	0.7
Input image size	640

**Table 2:** Series Model Comparison Metrics Table.

Model	GFLOPS	Precision	Recall	mAP50	mAP50-95
YOLOv10n	8.2	0.827	0.858	0.869	0.627
YOLOv11n	6.3	0.903	0.864	0.899	0.684
Our	6.2	0.917	0.882	0.897	0.684

**Table 3:** Experimental detection results of introducing other convolutional models.

Model	GFLOPs	Precision	Recall	mAP50	mAP50-95
YOLOv11n	6.3	0.903	0.864	**0.899**	0.684
+DS-Neck	5.9	0.881	0.843	0.871	0.658
+AK-Neck	6.2	0.916	0.845	0.882	0.653
+DW-Neck	5.9	0.887	**0.885**	0.892	0.679
+Deform-Neck	**6.2**	**0.916**	0.871	0.895	**0.684**

**Table 4:** Ablation experiment for the effectiveness of module combination.

Method		AP		GFLOPS	Precision	Recall
LSKA	Deform-Neck	Scratch	Damage			
Baseline		0.918	0.904	6.3	0.903	0.864
√		0.901	0.914	**5.9**	0.889	**0.895**
	√	**0.93**	**0.943**	6.2	0.916	0.871
√	√	0.926	0.94	6.2	**0.917**	0.882

## Data Availability

The authors confirm that the data supporting the findings of this study are available within the article.
